# Long‐term effects of glucocorticoid excess on the brain

**DOI:** 10.1111/jne.13142

**Published:** 2022-08-18

**Authors:** Alies J. Dekkers, Jorge Miguel Amaya, Merel van der Meulen, Nienke R. Biermasz, Onno C. Meijer, Alberto M. Pereira

**Affiliations:** ^1^ Department of Medicine, Division of Endocrinology, Pituitary Center and Center for Endocrine Tumors Leiden University Medical Center Leiden The Netherlands; ^2^ Department of Medicine, Center for Endocrine Tumours Leiden Leiden University Medical Center Leiden The Netherlands; ^3^ Department of Endocrinology & Metabolism Amsterdam UMC (AMC) Amsterdam The Netherlands

**Keywords:** cognition, corticosteroid, Cushing, glucocorticoid, neuroimaging, neuropsychology

## Abstract

The metabolic and cardiovascular clinical manifestations in patients with Cushing's syndrome (CS) are generally well known. However, recent studies have broadened the perspective of the effects of hypercortisolism, showing that both endogenous and exogenous glucocorticoid excess alter brain functioning on several time scales. Consequently, cognitive deficits and neuropsychological symptoms are highly prevalent during both active CS and CS in remission, as well as during glucocorticoid treatment. In this review, we discuss the effects of endogenous hypercortisolism and exogenously induced glucocorticoid excess on the brain, as well as the prevalence of cognitive and neuropsychological deficits and their course after biochemical remission. Furthermore, we propose possible mechanisms that may underly neuronal changes, based on experimental models and in vitro studies. Finally, we offer recommendations for future studies.

## Graphical abstract

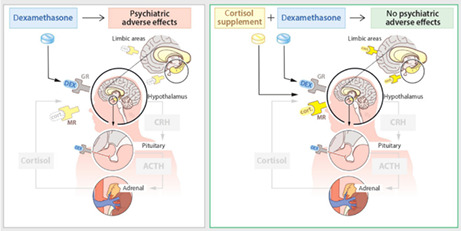

Preventing neuropsychiatric adverse effects of dexamethasone with co‐administration of cortisol. Dexamethasone suppresses the HPA‐axis and thereby the adrenocortical cortisol production, leaving cortisol‐preferring MRs unoccupied. Re‐activating the MR using cortisol might reduce or prevent neuropsychiatric adverse effects of dexamethasone treatment via activation of brain MR. ACTH, adrenocorticotropic hormone; CRH, corticotrophin‐releasing hormone; GR, glucocorticoid receptor; HPA, hypothalamus‐pituitary‐adrenal; MR, mineralocorticoid receptor

## 1 ENDOGENOUS HYPERCORTISOLISM AND EXOGENOUSLY INDUCED GLUCOCORTICOID EXCESS

Cushing's syndrome (CS) develops as a result of chronic glucocorticoid excess and can be the consequence of either endogenous hypercortisolism or corticosteroid use (exogenously induced glucocorticoid excess). Cortisol acts via two types of receptors: the mineralocorticoid (MR) and the glucocorticoid (GR) receptors. The mineralocorticoid hormone aldosterone can also bind MRs but is outcompeted by cortisol for binding, unless these naturally occurring glucocorticoids are enzymatically degraded by 11β‐hydroxysteroid dehydrogenase type 2. In the brain, most MRs are cortisol‐preferring rather than ‘aldosterone selective’, with the exception of a few discrete neuronal populations in the nuclei tractus solitarii, the ventromedial hypothalamus and circumventricular organs.^1^

The widespread distribution of the GR outside the central nervous system explains the typical syndrome that develops after chronic excess cortisol exposure affecting metabolic, cardiovascular, immunological, reproductive, psychological and cognitive functioning during active disease. After biochemical remission, many symptoms and quality of life (QoL) improve, but significant morbidity and decreased QoL persist compared to healthy controls and patients treated for other pituitary adenomas.^2^ In addition to the cardiovascular and metabolic morbidity of hypercortisolism, patients experience psychological and cognitive impairments.^3^ The cause of psychological morbidity in CS patients is likely multifaceted, and the effects of glucocorticoid excess, chronic disease per se, and cardiovascular comorbidities are likely involved.^2^, ^3^ Conversely, altered hypothalamic‐pituitary‐adrenal (HPA) axis functioning has also been related to different psychiatric disease and cognitive impairment.^4^

### 1.1 Glucocorticoids and exogenously induced glucocorticoid excess

**FIGURE 1:**
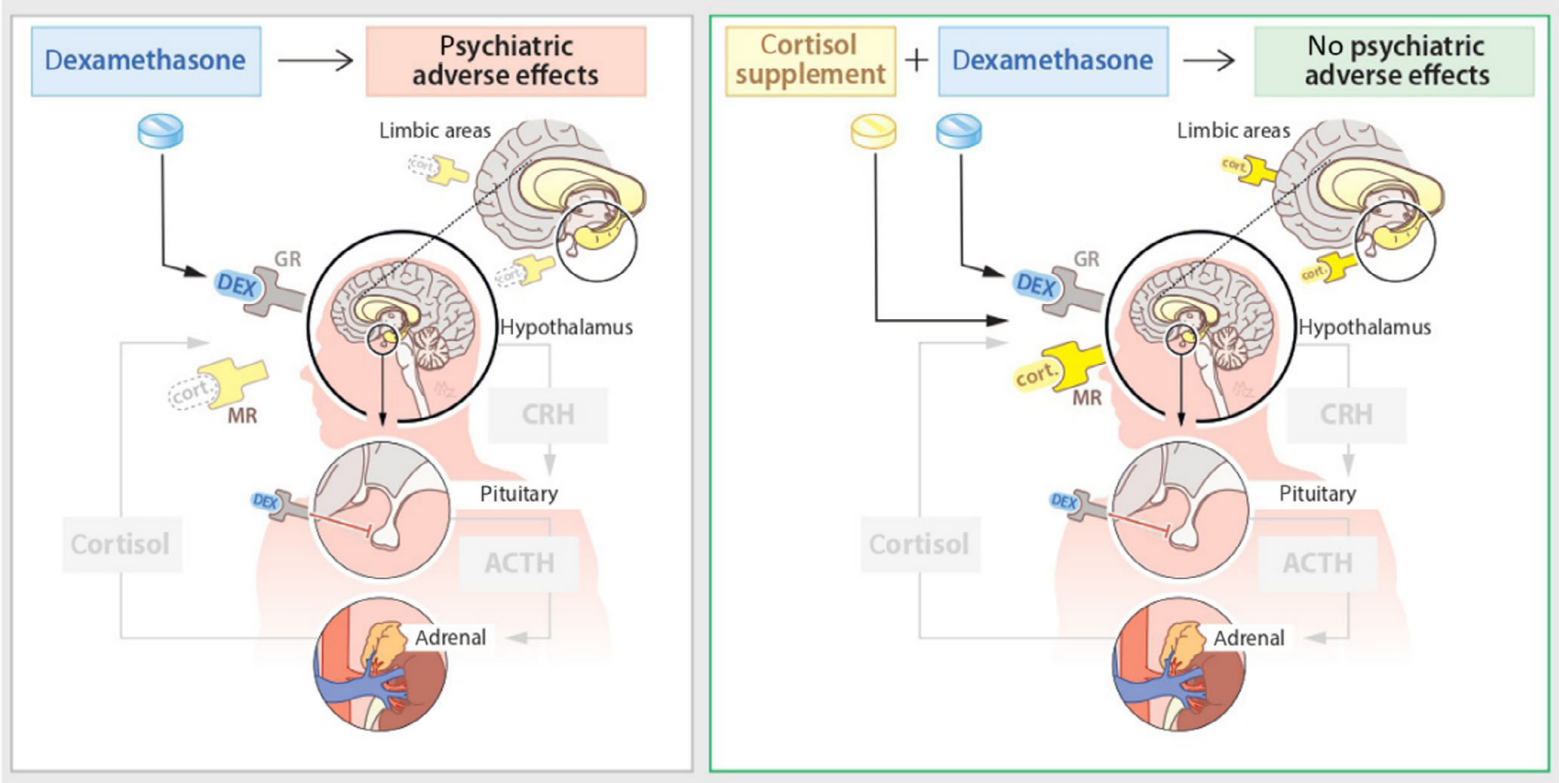
The effects of glucocorticoids and their potential effects on glucocorticoid (GR)/mineralocorticoid (MR) occupation in the brain. ACTH, adrenocorticotropic hormone; CRH, corticotropin‐releasing hormone; DEX, dexamethasone; cort., cortisol; GR, glucocorticoid receptor; MR, mineralocorticoid receptor. Adapted with permission from De Koning *et al*. 2021.^11^

Synthetic glucocorticoids (e.g., corticosteroid therapy) are synthetic analogues of endogenous steroids and act via the GR. Glucocorticoids have strong anti‐inflammatory properties and are therefore prescribed for various autoimmune, allergic and inflammatory diseases. Besides the therapeutic effects, glucocorticoids also exert side effects through excessive stimulation of the GR and affect the activity of the endogenous HPA axis. For example, synthetic glucocorticoids disrupt circadian adrenocorticotropic hormone (ACTH) and cortisol release, and this contributes to cardiovascular complications, mood disorders or insomnia.^5^ These side effects may occur during short or chronic therapy,^6^, ^7^, ^8^ and may develop as soon as glucocorticoid therapy is initiated.^9^ Because synthetic glucocorticoids bind more potently to GR than MR, relative under‐activation of MR as a consequence of HPA axis suppression may also contribute to symptomatology and side effects.^10^ This may aggravate neuropsychological dysfunction, the concept of which is currently under investigation in a randomized controlled trial evaluating the effects of physiological hydrocortisone replacement doses vs. placebo in patients with brain tumours who are treated with the potent synthetic corticosteroid dexamethasone^11^ (Figure 1).

## 2 GLUCOCORTICOID EXCESS, THE BRAIN, PSYCHOLOGICAL/COGNITIVE SYMPTOMS AND QOL

The effects of hypercortisolism on the brain have been described for decades, with early studies showing cerebral atrophy and enlarged ventricles in patients with both endogenous or exogenous glucocorticoid excess.^12^, ^13^ More specifically, glucocorticoid excess is related to structural and functional changes in the limbic system and cortex, which potentially underly cognitive impairments and neuropsychiatric symptoms in patients with CS. Altered brain structure and functioning may be a direct consequence of the effects of glucocorticoids on the MR and GR expressed by neurons in different brain areas. Furthermore, they may modulate the brain indirectly through their effect on the immune system (e.g., through astrocytes, microglial cells), metabolism, sleep and other hormones, with effects that may persist after remission or cessation of glucocorticoids.^14^

**FIGURE 2:**
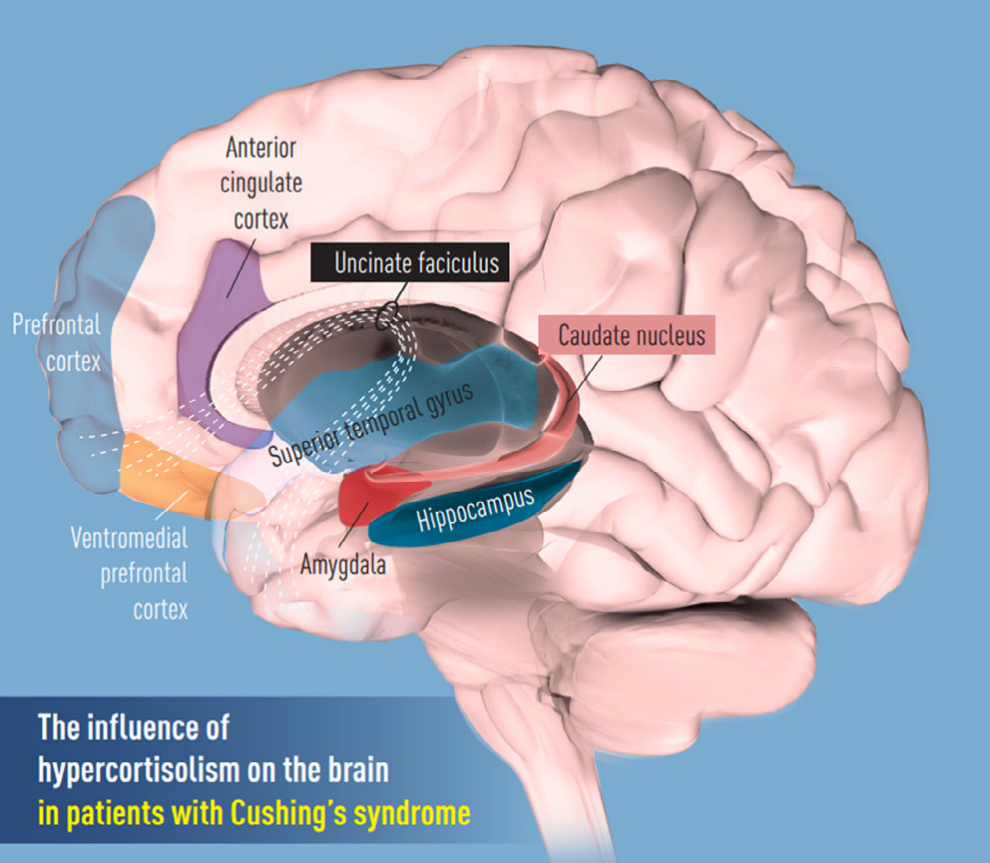
The influence of hypercortisolism on the brain. Adapted with permission from the Journal of Internal Medicine^26^

This review discusses the effects of hypercortisolism on the brain, the effect of hypercortisolism on cognitive functioning and neuropsychological symptoms, the reversibility of changes after biochemical remission or cessation of glucocorticoids, and the similarities between the effects of endogenous hypercortisolism and exogenous glucocorticoid excess on the brain (Figure 2).

## 3 IMAGING STUDIES IN HUMANS

### 3.1 First studies/autopsies

The effects of chronic glucocorticoid exposure on the human brain were first described in the 1950s in an autopsy report of a deceased patient with Cushing's disease (CD), who had dilated lateral and third ventricles and a relatively low brain weight.^15^ Twenty years later, another study showed a high incidence of cerebral and cerebellar cortical atrophy in Cushing's patients.^13^ In the modern era, with advancements in imaging techniques, we and others have been able to study structural and functional brain changes in more detail. Structural and functional magnetic resonance imaging (MRI) can be used to measure microstructural integrity, including diffusional tensor imaging to display anisotropic properties (i.e., diffusion of water) of tissue. Proton magnetic resonance spectroscopy (H‐MRS) displays metabolic state in neurons through measuring neurotransmitter concentrations in neurons.

## 4 IMAGING STUDIES IN PATIENTS WITH CD: ACTIVE CS VERSUS CS IN REMISSION

### 4.1 Structural MRI

#### 4.1.1 Gray matter: cerebral (sub)cortical atrophy

Multiple studies have reported global cerebral atrophy and volumetric reductions in specific brain areas in patients with active CS. Decreased gray matter volumes in limbic structures (i.e., the hippocampus, amygdala and anterior cingulate cortex [ACC]) have been found in patients with active CD, which only partially restore after remission.^16^, ^17^, ^18^, ^19^ These volumetric changes were more severe in patients with higher cortisol levels^16^, ^17^, ^19^ or longer disease duration.^19^, ^20^ In addition, one recent study found decreased gray matter densities in areas that are part of the ‘rich club network’, a network comprising brain areas that are simultaneously activated during cognitive tasks, located in the superior parietal lobe, superior frontal cortex, precuneus, putamen, thalamus and hippocampus. Anatomical changes in rich club nodes restored 3 months after successful surgical treatment.^21^ Interestingly, an increased volume in the bilateral nucleus caudatus was found in patients with CD and was positively related to disease duration.^19^ Altogether, patients with active CS or CS in remission show mostly decreased gray matter volumes in different brain areas. However, it is possible that compensatory neurogenesis results in increased gray matter volume.

#### 4.1.2 White matter integrity (diffusional tensor imaging)

In addition to cortical and subcortical atrophy, diffuse white matter hyperintensities (i.e., loss of white matter fibers) are more prevalent in patients with CS compared to healthy controls, and have the potential to reverse after correction of hypercortisolism.^18^ Altered white matter microstructure, particularly in the frontal lobe and limbic system, has been found in patients with active CS or CS in remission,^22^, ^23^, ^24^, ^25^ and correlated with worse depressive symptoms and processing speed.^23^ More specifically, white matter microstructure had decreased fractional anisotropy and increased diffusivity, reflecting myelin and/or axonal damage.^23^

### 4.2 Functional MRI (fMRI)

#### 4.2.1 Resting state fMRI

Besides structural imaging techniques, a limited number of studies also employed other imaging modalities to investigate brain function in this patient population. Resting state fMRI studies of patients with active CD showed increased spontaneous activity in the posterior cingulate cortex and left prefrontal cortex (PFC)^26^ and impaired functional connectivity in areas of the rich‐club network (e.g., precuneus, cingulum and inferior temporal lobe).^21^ Paradoxically, interconnectedness between rich‐club nodes and integratory processing were enhanced, as reflected by higher global efficiency and shorter path lengths.^21^

After remission, changes in rich‐club structures can restore 3 months after successful surgical treatment.^21^ Increased activity in the medial temporal lobe, hippocampus and PFC may improve compared to active disease, but remain higher compared to healthy controls.^26^ Also, increased connectivity between the default mode network (DMN) and superior left lateral occipital cortex may persist, even years after remission.^26^ The DMN comprises different brain areas that are simultaneously activated during self‐referential processes and are deactivated during external or cognitive processes, and altered activity may impair cognitive tasks, especially those involving behavior suppression or control.^27^ Increased activity in the DMN during a resting state MRI may enhance one's emotional awareness towards negative feelings (i.e., negative cognitive bias) and result in anxiety or depression.^28^

#### 4.2.2 Task‐related fMRI

During memory encoding tasks, patients with active CD had increased brain activity in the amygdala and hippocampus,^17^ which may indicate enhanced encoding of fearful memories and result in more anxiety. An fMRI study comparing participants with high or low anxiety traits showed increased functional connectivity between the subgenual ACC and bilateral amygdala, which was related to higher morning cortisol increase. Higher anxiety was also related to enhanced negative emotions encoding and impaired positive emotions encoding, suggesting that increased cortisol may alter emotional processing and result in negative cognitive bias and more anxious or depressive feelings.^29^ Furthermore, during a facial expression identification task, patients with CD had decreased activity in the left anterior‐superior temporal gyrus, which likely reflects impaired facial expression identification and emotion processing.^30^

One study found that remitted CD patients had decreased functional activity in the medial prefrontal cortex during facial expression processing and memory tasks. These functional impairments may reflect broader cognitive deficits (e.g., working memory) observed during active disease and after remission,^17^ a finding that is supported by the increased reaction time in remitted CD patients to identify emotional faces correctly.^30^ Furthermore, impaired connectivity between the ventromedial PFC and posterior cingulate cortex were observed during facial expression processing. Taken together, the fMRI findings show altered neuronal processing in patients with active or remitted CD, particularly in regions in or connecting to the PFC and limbic structures, likely corresponding with impaired emotional processing and cognitive functioning.

### 4.3 H‐MRS

Lastly, a few studies investigated brain metabolism using H‐MRS, showing altered brain metabolites in patients with CS. Hippocampal volumes in patients with CS in remission were normal, but H‐MRS imaging revealed decreased *N*‐acetyl‐aspartate (NAA) and increased glutamine in bilateral hippocampi compared to healthy controls. These changes may reflect reduced neuron density and impaired compensatory remodulation by micro glial cells or astrocytes.^31^ In addition, lower NAA and glutamate in the ventromedial PFC were found in patients with active CS and CS in remission. Disease duration was associated with lower NAA and increased anxiety, indicating that endogenous hypercortisolism impairs neuronal integrity in the ventromedial PFC, an area that is involved in experiencing positive or negative emotions, and enhances anxiety.^32^

## 5 EXOGENOUS GLUCOCORTICOID EXCESS

Although, in the context of glucocorticoid exposure, most imaging studies have been performed in patients with CS, some of these findings have also been described in patients using oral glucocorticoids medication.

Because exogenous glucocorticoids have different binding potency for the MR and GR than endogenous glucocorticoids, they may disturb the MR/GR occupation and alter brain structure and functioning.^11^

### 5.1 Structural MRI

Work by Brown et al.^33^ has demonstrated that chronic glucocorticoids use is associated with gray matter volumetric reductions in the hippocampus and amygdala, and that a 3‐day high‐dose course of hydrocortisone is already associated with a 1.69% decrease in hippocampal volume in healthy individuals, which was positively correlated with cortisol levels and resolved within 1 month after cessation.^34^ Reduced amygdala volume has been correlated with glucocorticoid therapy duration^33^ and changes were reversed by glutamate antagonists, suggesting that glucocorticoids may induce hyperexcitation of glutamate neurons in the amygdala.^35^

In children and adolescents with chronic glucocorticoids treatment, altered white matter microstructure has been observed in the uncinate fasciculus, a structure that regulates emotional processing through connecting the limbic system with prefrontal cortex.^36^ In patients with systemic lupus erythematodus, altered white matter structure correlated with cumulative glucocorticoids dosage and attentional deficits.^37^

### 5.2 fMRI

fMRI studies showed that short‐term glucocorticoid administration in healthy subjects decreased hippocampal activity on resting fMRI^38^ and reduced cerebral blood flow (i.e., compromising metabolic capacity) in the posterior medial temporal lobe during cognitive tasks.^39^

### 5.3 H‐MRS

One study investigated the effect of pharmacologically induced stress, using 54 mg of yohimbine and 10 mg of hydrocortisone. Stress impaired dorsolateral PFC glutamate release, indicating altered excitatory activity and reduced neuron metabolism. Also, subjects showed impaired working memory, suggesting that stress can limit metabolic capacity of neurons and efficient functioning.^40^ Another study showed that patients receiving chronic glucocorticoids therapy had lower hippocampal NAA levels and volume, reflecting compromised neuron viability.^38^

## 6 NEUROCOGNITIVE AND PSYCHOLOGICAL EFFECTS OF LONG‐TERM CORTISOL EXCESS

A higher prevalence of neuropsychiatric disease and lower QoL has been reported in both endogenous (i.e., CS) and exogenously induced (i.e., glucocorticoid therapy) hypercortisolism compared to healthy controls.^26^, ^39^ Psychiatric morbidity has been reported in up to 81% of patients with active CS,^26^ varying from affective symptoms, such as a depressed mood, to severe neuropsychiatric disease, including therapy refractory psychoses.^41^ We next discuss the prevalence of neuropsychological and cognitive deficits in patients with CS, different clinical manifestations, and the course of symptoms after biochemical remission. The findings are summarized in Table 1.

**TABLE 1:** Prevalence of cognitive and neuropsychological impairments in Cushing's syndrome (CS) and course after remission

Cognitive domain	Active CS		Course after > 6 months biochemical remission
	Objectively impaired	Subjectively impaired	
Learning and memory	83% memory impairments Impaired memory encoding and consolidation^42^, ^43^	78% memory deficits, forgetfulness^42^	Improved, but not normalized^42^, ^43^
Executive functioning	Various executive functioning deficits (i.e., concept formation, reasoning, decision making and error detection)^26^ 15% Cognitive flexibility deficits^42^ Impaired processing speed during longer lasting tasks^42^ Minor impairments in executive functioning^43^	39% impaired processing speed^42^	Slightly improved in most studies, but not normalized^26^, ^42^, ^43^
Attention and concentration	66% Concentration deficits^26^ 20%–40% Sustained attention deficits^42^	94% attention or concentration difficulties^42^	Improved, but not normalized^43^
Visuospatial	30% impaired line orientation^42^ Impaired visuospatial organization^43^	NA	Improved or resolved over the long term^43^
Language	Minor language impairments^43^ Word finding/fluency was not impaired^42^	67% word finding difficulties^42^	Improved or resolved^43^
Affective	60% Somatization^42^ 60%–70% Depression^26^, ^42^ 12%–79% Anxiety^26^, ^42^ 53% Panic disorder^26^ 4% (Hypo)mania^26^	50% Irritability^42^ 40% Depressive^42^ 40% Anxiety^42^ 33% Emotional lability^42^	Improved or recovered in most cases, but persistent depressive symptoms are present in up to 25%^26^
Neuropsychiatric disease	8% Psychotic disease^26^	NA	NA
Other	50% Disturbed sleep^26^	NA	NA

Abbreviations: CS, Cushing's syndrome; NA, not applicable.

## 7 COGNITIVE AND PSYCHOLOGICAL FUNCTIONING

### 7.1 Learning and memory

Over 80% of patients with active CD have memory impairments amongst different domains.^17^, ^26^ A comprehensive neuropsychological assessment study found that learning and memory related to visuospatial and linguistic information processing were mostly affected. Memory impairments were profound during encoding‐related tasks (i.e., new information), whereas memory retention and retrieval (i.e., recall after repetition) were preserved. Intriguingly, objective memory impairments may be present in 30% of cases, whereas 80% of patients experience subjective impairments,^42^ suggesting that neuropsychological assessment may lack sensitivity to detect minor, but individually relevant, impairments. Memory deficits are proposed to be related not only to temporal lobe atrophy, but also indirectly to changes in the frontal lobe.^16^

### 7.2 Executive functioning

This includes working memory, error detection, concept formation, information integration and decision making. In patients with active CS, impaired processing speed, concept formation, error detection, cognitive flexibility (i.e., task switching) and decision making have been observed in approximately 15% of patients. Again, subjective impairments were more prevalent (40%). Cognitive fatigue and/or less efficient energy consumption may affect processing speed and increased error rates^26^, ^43^ Executive functioning impairments are probably related to frontal lobe atrophy.^26^

### 7.3 Attention and concentration

Attentional processes occur throughout the brain, depending on the nature of the task (e.g., selective or divided, top‐down or bottom‐up based), including cortical areas in the frontal lobe, parietal lobe and subcortical structures, such as the thalamus. It is therefore important to distinguish between types of attentional deficits. In general, concentration and attentional impairments may be present in 30%–60% of patients with active CS.^26^, ^42^ More specifically, selective attention impairments have been found, particularly during tasks requiring alerting, orienting or divided attention.^44^ Sustained attention deficits were also reported in 20%–40% of patients with active CS, whereas simple attention functioning was preserved.^42^ Subjectively, 94% experienced impaired attention.^42^ The various attentional deficits may overlap with impairments in executive functioning or memory. How attentional deficits are related to neurobiological processes is still unknown.

### 7.4 Visuospatial, perception and motor functions

Approximately 30% of patients with active CS have moderate visuospatial impairments,^26^, ^42^, ^45^ which included visuospatial construction and orientation deficits that were associated with higher baseline ACTH.^42^ Altered visual processing, proprioception and memory may contribute to visuospatial impairments and could be linked to neuroanatomical changes in the occipital or temporal lobe.^43^ However, the extent of visuospatial impairments and their neurobiological substrates also need further exploration.

### 7.5 Language

Vocabulary, word finding and verbal fluency may be impaired in patients with active CS, whereas some studies did not find linguistic deficits.^42^, ^43^ However, objective measurements may fail to capture subjective verbal impairments, which may be present in more than two‐thirds of cases.^42^

### 7.6 Affective symptoms

Are the most common neuropsychiatric manifestations during active disease. At diagnosis, 60%–65% of patients have an affective disorder, mostly depression or anxiety, but mania has also been reported.^26^, ^42^ Other mood‐related symptoms include higher levels of somatization, irritability, negative mood and emotional lability.^42^ After remission, the mood symptoms persist and correlate with structural changes in the anterior cingulate gyrus and disease duration.^20^

### 7.7 Psychoses

These may present in 8% of patients with active CS.^26^ Although psychoses are relatively rare, they may be therapy refractory, resulting in recurrent psychoses and significant morbidity.^41^

## 8 COURSE OF SYMPTOMS AFTER REMISSION

Generally, cognitive deficits and neuropsychiatric symptoms improve after correction of glucocorticoid excess, although they remain impaired compared to healthy controls,^26^, ^42^ which may be related to the observed long‐term structural and functional brain changes after excess glucocorticoid exposure. For example, depressive symptoms improve in 70%–75% of remitted patients, but symptoms persist in one‐quarter of them, even after years in remission.^26^ How other aspects of cognitive functioning develop after remission remains unclear, but, overall, it appears that most cognitive functions improve towards normal.^26^ Improvements may occur as soon as within 1 week after normalized glucocorticoid excess.^26^ However, because some aspects of cognitive functioning fail to normalize, special and continuous attention for these specific aspects might improve and possibly normalize QoL.

## 9 EXOGENOUS GLUCOCORTICOID EXCESS AND NEUROPSYCHOLOGICAL SYMPTOMS

It is well recognized in clinical practice that patients treated with glucocorticoids develop the same physical characteristics as patients with endogenous Cushing's syndrome, although this is much less apparent for the neuropsychological phenotype. This is at least partly a result of overlapping symptomatology attributed to the underlying disease for which the glucocorticoids are prescribed. In line with the effects of endogenous hypercortisolism, exogenous glucocorticoid administration is also associated with neuropsychological symptoms and cognitive impairments. These side effects often develop within a few days after treatment initiation and resolve after a few days upon cessation of glucocorticoids.^6^ The likelihood of occurrence increases both with the duration of treatment and with increasing dose.^38^, ^39^ Initiation of glucocorticoids also showed a dose‐dependently increased risk for severe neuropsychiatric manifestations, including suicide attempts and confusion, as described in more detail by Judd *et al*.^39^ In addition to neuropsychological symptoms, impaired memory and executive functioning have also been observed.^6^ It appears that memory consolidation, as opposed to memory acquisition, is especially affected by glucocorticoids, considering that immediate‐recall tasks were preserved.^6^ Sleep disturbances (insomnia) are highly prevalent both after short‐ and long‐term use, and occur already after treatment with low doses.^6^, ^39^ Little is known about the extent of reversibility of psychopathology and cognitive dysfunction though the general assumption is that most changes are reversible upon treatment cessation.^6^

## 10 EVIDENCE FROM EXPERIMENTAL MODELS

Historically, much work on glucocorticoid excess on the brain was performed in relation to chronic psychosocial stress, and most of this work focused on neurons. Early work focused on the hippocampus, and emphasized the vulnerability of neurons to excitotoxicity after stress and glucocorticoid exposure.^46^ It also became clear that the neuronal architecture changes after chronic stress: the complexity of dendritic trees is reduced in the hippocampal CA3 area,^47^ yet increases in some striatal areas.^48^ Although such changes could be linked to changed behavioral reactivity and sometimes were associated with changes in (hippocampal) volume, it is unclear whether they are responsible for overall changes in gray matter. Moreover, the ‘neurocentricity’ of this work perhaps neglected the presence of GR in all other cell types in the brain. Other lines of research investigated the effect of glucocorticoids on neurodegenerative processes through the GR located on astrocytes and microglia cells, although the readouts of these studies have been rather remote from those in Cushing's patients.^49^

More recently, the group of Antoine Martinez developed mouse models for adrenally driven endogenous CS, including the so‐called AdKO mouse model. By introducing a human mutation in the protein kinase A pathway in adrenocortical cells, these mice develop full‐blown Cushing's symptomatology with metabolic changes that even include the ‘buffalo hump’. Of note, these mice also show quite extensive changes in relative brain volumes, as evaluated by MRI.^50^ In these data, changes in white matter areas are very prominent. Indeed, immunohistochemistry showed a reduction in markers for oligodendrocytes, as well as active astrocytes and microglia.^50^ These findings were later extended by mRNA measurements. Overall, both neuronal and glia cell populations are affected by the chronic hypercortisolemia.^50^, ^51^ The extensive changes in oligodendrocyte markers suggest that white matter changes are the consequence of direct targeting of the GR in these cells, which indeed express high levels of GR.^52^

Indeed, Sgk1 mRNA expression in oligodendrocytes is highly responsive to stress or circadian glucocorticoid peaks.^53^ Two main features of mature oligodendrocytes, myelin basic protein expression and myelination index, decreased after exposure to high concentrations of glucocorticoids.^54^ Early studies reported that glucocorticoid treatments in rodents reduced myelin thickness and amount.^55^ Myelination impairment was also observed in experimental prenatal administration of high doses of glucocorticoids, and both exogenous glucocorticoids and chronic stress reduced remyelination in multiple sclerosis animal models.^56^

It is important to note that not all psychiatric symptoms of Cushing's patients need to be linked to structural changes that can be found by MRI. For example, AdKO mice showed strongly induced expression of FKBP5 and, in the amygdala and hippocampus, CRH.^51^, ^52^ In particular, the latter may be directly linked to anxiety and hyperarousal. It will be interesting to see to what extent the changes in these mouse models will be reversible after adrenalectomy or treatment with, for example, GR antagonists.

## 11 CURRENT STATUS AND FUTURE PERSPECTIVES

Patients with CS show structural and functional brain changes, particularly in the prefrontal cortex and limbic system, in the presence of neuropsychological and cognitive dysfunction. Neurobiological changes may occur either directly through altered stimulation of both MR/GR in the brain or through the effect of glucocorticoids on other physiological functions, such as metabolism, the immune system and sleep. After biochemical remission or cessation of glucocorticoids, changes improve, but not all changes are completely reversible on the presence of long‐term impaired QoL. Altogether, the findings indicate that early biochemical control and restraint use of glucocorticoids may prevent altered brain functioning. Because of the complexity and heterogeneity of impaired physical and psychological functioning after treatment of CS, an additional focus on amelioration of mental well‐being is advocated to improve QoL. This can be accomplished through inclusion of psychologists and psychiatrists in the multidisciplinary team of patients treated for CS at short‐ and long‐term follow‐up. Importantly, however, formal assessment methods likely underestimate the prevalence of subjective complaints and the burden of neuropsychological symptoms. Noteworthy, a systematic, sensitive, periodical assessment is currently not available, thereby underestimating the true prevalence and severity of neuropsychological symptoms. It is also likely that glucocorticoid replacement strategies better mimicking the physiological, circadian cortisol release may improve cognitive and neuropsychological symptoms in patients with adrenal insufficiency.^57^

Despite the accumulating evidence of glucocorticoid‐related adverse effects on the brain and neuropsychological functioning, no effective treatment strategies have yet been developed. One study in rodents found that treatment with an NMDA antagonist attenuated glucocorticoid‐induced hippocampal atrophy and memory deficits,^58^ indicating that glucocorticoid‐induced brain changes are rather caused by multiple factors than a singular mechanism. It is likely that indirect effects of glucocorticoids on physiological functions (e.g., cardiovascular, sleep and immunological) and individual factors (e.g., epigenetics, concomitant hypopituitarism, coping strategies and lifestyle) contribute to impaired brain functioning. Future studies are needed to relate cortisol excess with clinical outcomes and molecular mechanisms (which factors predispose specific brain areas/cells to glucocorticoids vulnerability, e.g., using the Allen Human Brain Atlas [https://human.brain-map.org/]) and to investigate to what extent changes are reversible. This will ultimately improve our understanding of the effects of glucocorticoids on the brain and cognitive functioning and facilitate the development of interventions preventing or recovering such changes. Of note, treatment with mifepristone was able to reverse long‐term consequences of severe stress in rodents.^59^ Also, newer and more selective GR antagonists and selective modulators are being developed, which may also be of benefit in active or remitted CD.^60^ Comprehensive neuropsychological assessment, imaging techniques and altered gene expression patterns may be critical. To improve glucocorticoid‐induced changes, a multifaceted approach targeting both direct and indirect effects of glucocorticoids will probably be required to optimize outcomes and QoL.

This article is part of an update series on the diagnosis and treatment of Cushing's syndrome.^61^, ^62^, ^63^, ^64^, ^65^, ^66^, ^67^, ^68^, ^69^, ^70^, ^71^, ^72^, ^73^, ^74^, ^75^, ^76^, ^77^

## AUTHOR CONTRIBUTIONS

**Alies Juliëtte Dekkers:** Writing – original draft. **Jorge Miguel Amaya:** Writing – review and editing. **Merel van der Meulen:** Writing – review and editing. **Nienke R Biermasz:** Writing – review and editing. **Onno C. Meijer:** Writing – original draft; writing – review and editing. **Alberto M. Pereira:** Methodology; writing – original draft; writing – review and editing.

## CONFLICTS OF INTEREST

The authors declare that they have no conflicts of interest.

### PEER REVIEW

The peer review history for this article is available at https://publons.com/publon/10.1111/jne.13142.

## Data Availability

Data sharing is not applicable to this article as no new data were created or analyzed in this study.
